# Performance Analysis of Single-Well Enhanced Geothermal System for Building Heating

**DOI:** 10.1155/2020/7927052

**Published:** 2020-07-13

**Authors:** Haijun Liang, Xiaofeng Guo, Tao Gao, Lingbao Wang, Xianbiao Bu

**Affiliations:** ^1^SINOPEC Star Petroleum Co., Ltd. (Sinopec Star), Beijing 100083, China; ^2^China National Research and Technology Center of Geothermal Energy, Beijing 100083, China; ^3^Guangzhou Institute of Energy Conversion, CAS, Guangzhou 510640, China

## Abstract

Deep borehole heat exchanger (DBHE) technology does not depend on the existence of hot water reservoir and can be used in various regions. However, the heat extraction from DBHE can hardly be improved due to poor thermal conductivity of rocks. Here, a single-well enhanced geothermal system (SWEGS) is proposed, which has a larger heat-exchange area of artificial reservoir created by fracturing hydrothermal technology. We find that, due to heat convection between rocks and fluid, the extracted thermal output for SWEGS is 4772.73 kW, which is 10.64 times of that of DBHE. By changing the injection water temperature, volume flow rate, and artificial reservoir volume, it is easy to adjust the extracted thermal output to meet the requirement of building thermal loads varying with outdoor air temperature. Understanding these will enable us to better apply SWEGS technology and solve the fog and haze problem easily and efficiently.

## 1. Introduction

In recent years, one of the greatest challenges in China is the fog and haze problem. About 83% of space heating areas in north China use coal, and annual coal consumptions are 400 million ton-coal-equivalent where decentralized coals in rural areas account for half of total consumptions [1]. A coal-based heating system contributes significantly to air pollution in winter. There is thus an urgent need for clean energy building heating technology at the present stage. Geothermal energy has showed significant potential as renewable energy resource because of its low environmental impact, low greenhouse gas emissions, and technical feasibility [2, 3].

**Figure 1:**
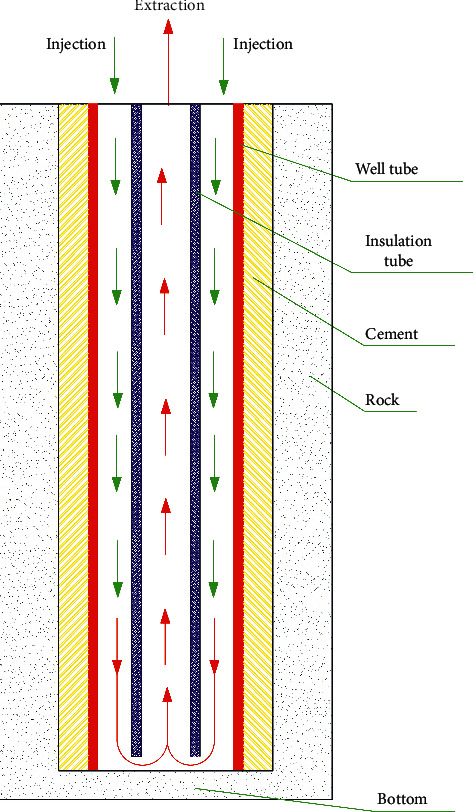
Structure diagram of DBHE.

**Figure 2:**
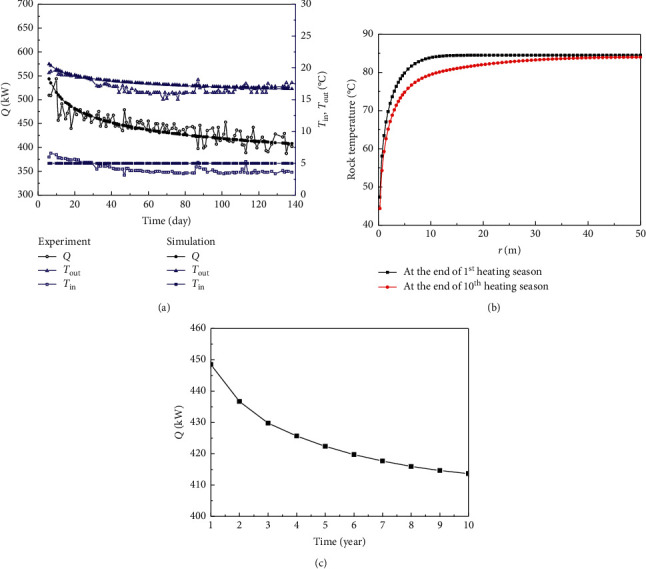
(a) Experimental data and simulation results [17, 18]. (b) Variation of rock temperature with radial distance at the end of first and tenth heating seasons with a depth of 2500 m. (c) Variation of the annual mean extracted thermal output with time. The symbol Q represents the extracted thermal output from DBHE, T_in_ is the injection water temperature, T_out_ is the extracted water temperature, and r is the radial distance from the well tube.

The hydrothermal system for geothermal heating relies heavily on the existence of rich hot water reservoir [3]. Moreover, the recharging problem also limits its application and promotion. Without the resource constraint and recharging problem, the technology of deep borehole heat exchanger (DBHE), as shown in Figure 1, is widely accepted in China, and it can be applied almost everywhere. Some researchers have focused on the utilization of geothermal energy acquired from DBHE for building heating [4–16]. Bu et al. carried out experimental and simulation studies of DBHE for building heating, and the research results are shown in Figure 2 [17, 18]. In Figure 2, DBHE is configured in a geothermal well with a depth of 2605 m, diameter 0.1778 m, bottomhole temperature 87.42°C, and average geothermal gradient 27.8°C/km. The volume flow rate is about 30 m^3^/h throughout the experimental process. It is obvious from Figure 2(a) that *Q* always decreases with time. The average extracted thermal output in the first heating season is 448.49 kW. As shown in Figure 2(b), the rock temperature decreases due to being cooled by the injection water, particularly the rocks close to well wall having the maximum temperature drop. The reason for this is that the heat loss from rocks to fluid cannot rapidly be compensated by heat conduction of rocks due to its poor thermal conductivity, resulting in a lower thermal output. It can thus be concluded that one of the effective means of improving the heat extraction from DBHE is to enhance the heat transfer of rocks, especially in near well bore zone. According to the principle of heat transfer, the heat transfer coefficient of the convection is far greater than that of the heat conduction. To conduct the convective heat exchange between fluid and rocks, the enhanced geothermal system (EGS) with artificial fractures created by the hydraulic fracturing was proposed [19, 20]. The conventional EGS has two or multiple vertical or directional wells, which causes the high drilling cost and thus limits its extensive applications [19, 20]. Moreover, the creation of the artificial fractures has a high risk, especially in the hard rock [21, 22]. In addition, the various physical and chemical processes take place in the geothermal reservoir during the process of fluid injection and extraction [23, 24].

**Figure 3:**
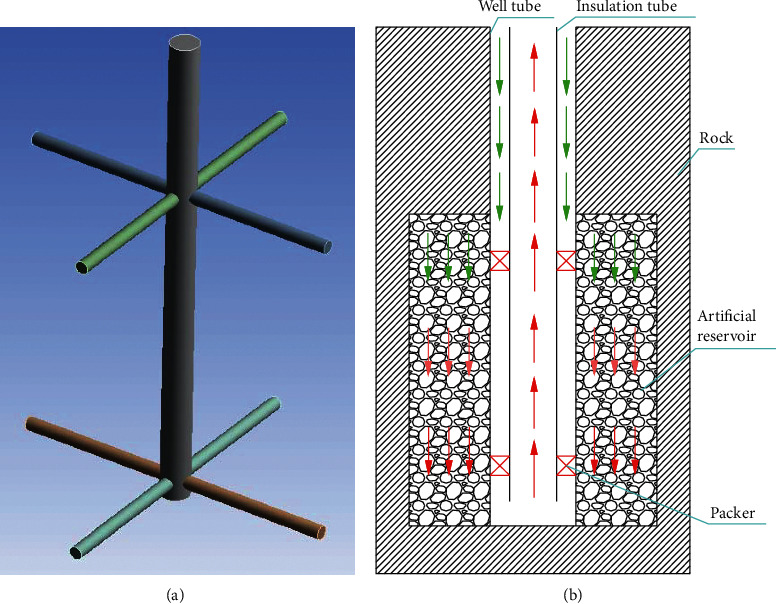
(a) Main well and multilateral wells. (b) Schematic of SWEGS.

To circumvent these problems, a single-well enhanced geothermal system (SWEGS), as illustrated in Figures 3(a) and 3(b), is proposed in this study. There are many hydrothermal systems in urban areas at the depth of less than 3000 m, which usually have small water output (usually less than 30 m^3^/h or even less). If the shallow depth hydrothermal system can be transformed into SWEGS for building heating, this not only has a good use of natural structural fissures in hydrothermal system but also reduces the risk and investment costs of EGS, and thus broadening the application range of EGS and effectively solving the fog and haze problem caused by winter heating using fossil energy. The artificial reservoir for SWEGS is created by two steps (1) drilling one main well and multilateral wells, as shown in Figure 3(a); (2) implementing hydraulic fracturing in the multilateral wells and transporting proppants to produce artificial reservoir. The function of the multilateral wells is to disperse fluid uniformly in the artificial reservoir and to make easy to fracture rocks. The common problems in both SWEGS and conventional EGS are, respectively, (1) the loss of circulating fluid; (2) the detection of formation stress distribution, fracture orientations, and tracer-swept volume; and (3) the risk of seismicity induced by hydraulic fracturing and so on. Compared to conventional EGS implemented in the hard rock, the creation of fractures is easy in hydrothermal system due to having many natural fractures. The achievements from SWEGS are expected to apply to conventional EGS and thus reducing its risk and costs. Although the spatial-temporal variation of fracture aperture during injection/production significantly influences the heat extraction from the reservoir, this is not the focus of this study [25]. The main aim of this study is to evaluate the performance of SWEGS so that more people know its performance and apply it for buildings heating to solve the fog and haze problem caused by using fossil energy.

## 2. Methods

### 2.1. Physical Model

The physical model of main well and multilateral wells and SWEGS is shown in Figures 3(a) and 3(b). In Figure 3, the main well has a depth of 2605 m, diameter 0.1778 m, bottomhole temperature 87.42°C, and average geothermal gradient 27.8°C/km. The density, specific heat, and thermal conductivity for rocks are, respectively, 2800 kg/m^3^, 0.92 kJ/kg/K, and 3.49 W/m/K. The rock physical property, well depth, and geothermal gradient for SWEGS are the same as those of DBHE. The diameter and length (distance from the main well) of multilateral wells are 0.08 m and 50 m, respectively. The artificial reservoir located at a depth of 2100–2600 m has a thickness of 500 m and a radius of 50 m. In general, there are more than four multilateral wells in the engineering projects.

Facing the engineering application, the following questions related to SWEGS technology need to be answered: (1) is the extracted thermal output of SWEGS stable and sustainable? (2) Can the extracted thermal output be easily adjusted as the variation of the building heating loads occurs? (3) What is the appropriate distance (well spacing) between two wells and appropriate thickness for artificial reservoir? These questions are hard to be addressed by the experimental studies due to the high cost. Alternatively, as a conventional method, numerical simulation can be used to analyze SWEGS performance in order to answer the above questions.

### 2.2. Model Equations

Assuming that the rocks enclosing the artificial reservoir are impermeable, that is, there is no fluid loss in the SWEGS. In addition, the influence of fluid-rock reaction is neglected during the process of injection and extraction fluid. The artificial reservoir is treated as equivalent to a porous medium with uniform porosity *ε* and permeability *K* [26–28]. Local thermal nonequilibrium between the porous rock matrix and fluid is considered, and thus, two energy equations are employed. The governing equations describing the conservation of continuity, momentum, and energy are formulated as the following [29, 30].

Fluid continuity equation is

(1) $$\begin{array}{c} \frac{\partial \left(\varepsilon \rho_{f}\right)}{\partial t}+\nabla \cdot \left(\rho_{f}u\right)=0. \end{array}$$

Fluid momentum equation is

(2) $$\begin{array}{c} \frac{\partial \left(\rho_{f}u/\varepsilon \right)}{\partial t}+\nabla \left(\rho_{f}\frac{u}{\varepsilon }\cdot \frac{u}{\varepsilon }\right)=-\nabla P+\nabla \left(\mu \nabla \cdot \frac{u}{\varepsilon }\right)-\frac{\mu }{K}u+\rho_{f}g. \end{array}$$

Energy equation in the porous rock matrix is

(3) $$\begin{array}{c} \frac{\partial {\left[\left(1-\varepsilon \right)\rho c_{p}T\right]}_{s}}{\partial t}=\nabla \cdot \left(\lambda_{s}^{eff}\nabla T_{s}\right)-h_{v}\left(T_{s}-T_{f}\right) \end{array}$$

Energy equation for heat transport in the fluid is

(4) $$\begin{array}{c} \frac{\partial {\left(\varepsilon \rho c_{p}T\right)}_{f}}{\partial t}+\nabla \cdot \left[{\left(\rho c_{p}T\right)}_{f}u\right]=\nabla \cdot \left(\lambda_{f}^{eff}\nabla T_{f}\right)+h_{v}\left(T_{s}-T_{f}\right), \end{array}$$

where **u**, *P*, *T*_*s*_, and *T*_*f*_ are the fluid velocity vector, fluid pressure, rock matrix temperature, and fluid temperature, respectively; *ρ*, *c*_*p*_, and *λ* with subscript “*f*” and “*s*”, respectively, indicate the density, specific heat capacity, and heat conductivity of fluid and rock matrix, respectively. The symbol *μ* is the viscosity of fluid. As annotated by the superscript, eff, the heat conductivity of fluid and rock matrix in the artificial reservoir is implemented with the effective forms, i.e., *λ*_*s*_^eff^=*λ*_*s*_(1 − *ε*)^1.5^ and *λ*_*f*_^eff^=*λ*_*f*_*ε*^1.5^, where *ε* denotes the artificial reservoir porosity [26]. The symbol *h*_*v*_ denotes the volumetric heat exchange coefficient between the porous rock matrix and fluid.

### 2.3. Initial and Boundary Conditions

The artificial reservoir is treated as equivalent to a porous medium, and its porosity and permeability is, respectively, 0.1 and 10^−12^ m^2^. The injection water temperature and volume flow rate are, respectively, 5°C and 50 m^3^/h. The heating time per year is 140 days, and the rest time is used for heat recovery.

It is assumed that the temperature of rocks is constant and not influenced by the fluid when *r* is larger than 200 m.

The heat exchange between the interface of artificial reservoir and surrounding rocks is given as follows:

(5) $$\begin{array}{c} h_{sf}\left(T_{s}-T_{f}\right)\left|{}_{r=50}\right.=\lambda_{s}\frac{\partial T_{s}}{\partial r}\left|{}_{r=50}\right., \end{array}$$

where *h*_*sf*_ is the convective heat transfer coefficient, which is calculated according to Dittus–Boelter formula [31].

(6) hsf=0.023λfRe0.8Pr0.4de,

where *d*_e_ is the hydraulic diameter, *d*_e_ = 100 m.

### 2.4. Numerical Method

Equations (1)–(4) together with the initial and boundary conditions are solved using MATLAB software. The algorithm of SIMPLE (Semi-Implicit Method for Pressure Linked Equation) is used to address the pressure-velocity coupling [32, 33].

## 3. Results and Discussion

**Figure 4:**
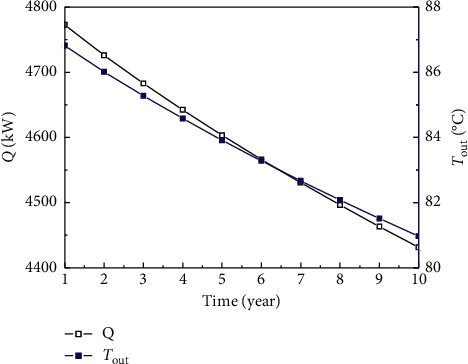
Variation of the annual mean extracted thermal output and extracted water temperature with time.

In Figure 4, the annual mean extracted thermal output for SWEGS ranges from 4431.38 to 4772.73 kW during ten heating seasons, with percentage reduction about 0.715% per year. The average *Q* for ten years is 4591.43 kW, which can provide heat for 131183.71 m^2^ buildings with a specific heat load of 35 W/m^2^ and can reduce carbon dioxide emissions by 13884.48 tons per heating season. The heating price is 4.32 USD/m^2^ each heating season at Qingdao in China, and thus, the total income from building heating is 566713.65 USD/year. In the first heating season, the annual mean extracted thermal output for SWEGS is 4772.73 kW, which is 10.64 times of that of DBHE. The extracted thermal output from SWEGS is much greater than that of DBHE, which is mainly caused by two reasons. First, the convective heat transfer is formed between fluid and rocks in the artificial reservoir, whose coefficient of heat transfer is much higher than that of heat conduction in DBHE. Second, large heat transfer area is created in the artificial reservoir by implementing hydraulic fracturing. The extracted heat is from two parts: one is heat convection between rocks and fluid in artificial reservoir, and the other is heat conduction from surrounding rocks to artificial reservoir. In Figure 2(c), the annual mean *Q* decreases drastically during the first few years, and then, the decreasing rate slows down gradually. The average *Q* for ten years in DBHE is 424.45 kW. The annual mean *Q* for DBHE is, respectively, 448.49 and 413.63 kW in the first and tenth heating seasons, which has a percentage change about 7.77%.

**Figure 5:**
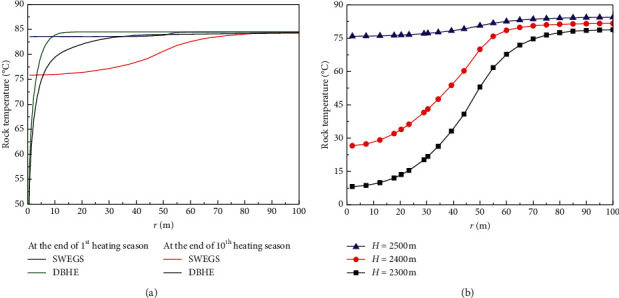
(a) Variation of rock temperature with radial distance at the end of first and tenth heating seasons with a depth of 2500 m. (b) Variation of rock temperature with different depth H at the end of the tenth heating seasons.

Figure 5(a) shows that there is a lower temperature gradient for the rocks in near well bore zone for SWEGS due to having a higher coefficient of convective heat transfer between fluid and rocks. Compared with undisturbed (initial) rocks temperature, the maximum rocks temperature drop for SWEGS is 8.63°C at a depth of 2500 m for ten years' operation, while it is 40.16°C for DBHE.

Figure 5(b) implies that more heat at the upper artificial reservoir is extracted by circulating water, as the injection water temperature is only 5°C. The temperature drop of artificial reservoir at a depth of 2400 m is big, while it becomes very small at a depth of 2500 m, which indicates that for ten years' operation, the cold front surface has not yet reached the depth of 2500 m, that is, to say, the service life for SWEGS is more than 10 years in terms of the reservoir thickness of 500 m, a multilateral well length of 50 m, the volume flow rate of 50 m^3^/h, and an injection water temperature of 5°C.

From Figure 5(b), the rocks temperature keeps unchanged (undisturbed) for *r* > 100 m at the end of tenth heating season, which indicates that the thermal influence radius (impact scope) is about 100 m for ten heating seasons. This leads us to conclude that in terms of the above parameters of the artificial reservoir shape and size and operation condition, the well spacing in the practical projects is recommended to be no less than 200 m with 10 years' operation period in order to avoid the mutual interference.

**Figure 6:**
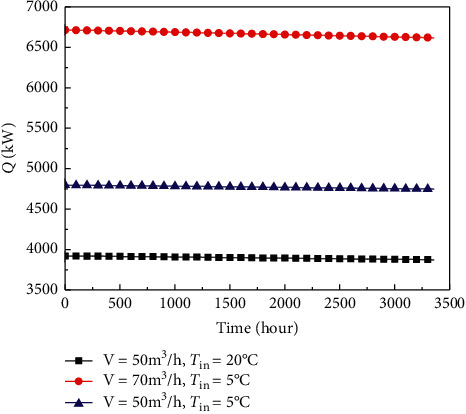
Variation of Q with T_in_ symbol and V.

**Figure 7:**
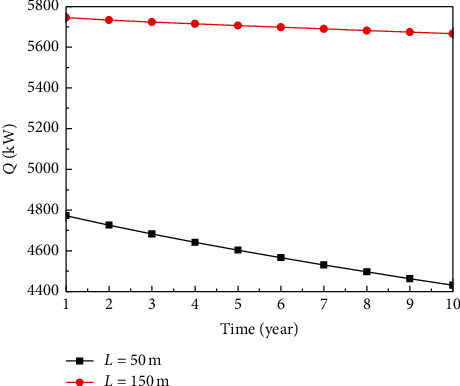
Variation of Q with multilateral well length.

From view point of heat transfer principle, three main methods can be used to enhance heat transfer between rocks and fluid. The first method is to improve heat transfer coefficient, which is reflected by improving the volume flow rate in Figure 6; the second method is to increase the heat transfer temperature difference, which is reflected by changing *T*_in_ in Figure 6; the third method is to increase heat exchange area, which is reflected by increasing the reservoir volume in Figure 7.

In Figures 6 and 7, the symbols V and *L*, respectively, represent the volume flow rate and the length of multilateral well. In Figure 6, the abscissa is the heating time at the first heating season (3360 h or 140 days). As evident in Figure 6, a higher volume flow rate will result in an increase in *Q*. A higher V will cause a higher convective heat transfer coefficient between rocks and fluid, thus leading to a greater *Q*. Generally speaking, the pump power has almost a cubic relationship with flow velocity. Consequently, an increase in V will require a rapidly increasing level of pump power. In the actual project, the pump power and *Q* should, therefore, be comprehensively considered so as to decide a reasonable V.

In addition, the temperature difference between rock and fluid increases with the decrease of *T*_in_ and thus leading to an increase in *Q*, which shows that the lower the *T*_in_, the higher the *Q*. Note that *T*_in_ should not be lower than zero temperature using water as working fluid; otherwise, it is easy to cause water to freeze. Therefore, the antifreeze liquid with good heat transfer performance and moderate cost can be considered as the injection fluid.

From Figure 6, *Q* can be adjusted by changing the injection water temperature and volume flow rate. In general, the outdoor air temperature has a great influence on the building heating loads. By adjusting the injection water temperature and volume flow rate, SWEGS can meet the requirement of building heating loads influenced by outdoor air temperature. Furthermore, the imbalance of the extracted thermal output among different heating seasons can also be adjusted by changing the injection water temperature and volume flow rate.

In Figure 7, the volume flow rate is 60 m^3^/h for artificial reservoir with *L* = 150 m, and other parameters are the same as those of the reservoir with *L* = 50 m. Figure 7 indicates that the larger the reservoir volume is, the slower the attenuation degree of *Q*. However, a large reservoir volume will result in a high engineering cost. Based on the above discussion, it is thus clear that the system performance, reservoir volume, and engineering cost should be comprehensively considered so as to decide their optimum matching relation.

In Figure 2(a), the temperature of extracted water from DBHE is lower than 20°C, which does not meet the demand of the heating temperature. Therefore, heat pump with user sides supply and return water temperature of 45°C and 40°C is needed in order to provide heat for building. While for SWEGS, the extracted water temperature is higher than 80°C, which can be used efficiently by adopting the model of cascade utilization (*T*_out_ > 60°C, direct heating by radiators; 45 < *T*_out_ < 60°C, heating by the fan coil units; 30 < *T*_out_ < 45°C, floor radiation heating; and *T*_out_ < 30°C, heating by heat pump).

## 4. Conclusions

Deep borehole heat exchanger (DBHE) technology does not depend on the existence of hot water reservoir and can be used in various regions. However, the heat extraction from DBHE can hardly be improved due to poor thermal conductivity of rocks. To conduct the convective heat exchange between fluid and rocks, the enhanced geothermal system (EGS) with artificial fractures created by the hydraulic fracturing was proposed. However, the EGS technology has a high drilling cost and a high risk for artificial fracturing. To circumvent these problems, a single-well enhanced geothermal system (SWEGS) is proposed.

There are many hydrothermal systems in urban areas at the depth of less than 3 km. In order to reduce the risk and the costs of EGS, the shallow depth hydrothermal system should be considered first. The shallow depth hydrothermal system has natural fractures, and it is easy to conduct hydraulic fracturing and stimulation to create new fractures and highly conductive zones and thus increasing the volume flow rate of SWEGS and effectively improving the heat-exchanging amount. Due to heat convection between rocks and fluid, the extracted thermal output for SWEGS is 4772.73 kW, which is 10.64 times of that of DBHE. Besides, by changing the injection water temperature, volume flow rate, and artificial reservoir volume, it is easy to adjust the extracted thermal output to meet the requirement of building thermal loads changing with outdoor air temperature. As a result, the fog and haze problem in China caused by winter heating using fossil energy can be effectively solved by applied SWEGS due to having many shallow depth hydrothermal systems in urban areas. In addition, the researchers should make great efforts to study the possibility or risk of seismicity induced by hydraulic fracturing during the process of creating artificial reservoir.

## Data Availability

The data used to support the findings of this study are included within the supplementary information file.

## Supplementary Materials

Supplementary Materials The raw data used to plot Figures 2, 4, 5, 6, and 7 are provided in the supplementary file. The symbol Q represents the extracted thermal output from DBHE, *T*_in_ is the injection water temperature, *T*_out_ is the extracted water temperature, and r is the radial distance from the well tube. The symbols V and L, respectively, denote the volume flow rate and the length of multilateral well. .
